# Two Sides of Emotion: Exploring Positivity and Negativity in Six Basic Emotions across Cultures

**DOI:** 10.3389/fpsyg.2017.00610

**Published:** 2017-04-20

**Authors:** Sieun An, Li-Jun Ji, Michael Marks, Zhiyong Zhang

**Affiliations:** ^1^Department of Psychology, Ashoka UniversitySonepat, India; ^2^Department of Psychology, Queen’s University,Kingston, ON, Canada; ^3^School of Psychological and Cognitive Sciences, Peking UniversityBeijing, China; ^4^Department of Psychology, New Mexico State University,Las Cruces, NM, USA

**Keywords:** emotion, affect, cognition, culture, dialectical thinking

## Abstract

We employ a novel paradigm to test whether six basic emotions (sadness, fear, disgust, anger, surprise, and happiness; Ekman, 1992) contain both negativity and positivity, as opposed to consisting of a single continuum between negative and positive. We examined the perceived negativity and positivity of these emotions in terms of their affective and cognitive components among Korean, Chinese, Canadian, and American students. Assessing each emotion at the cognitive and affective levels cross-culturally provides a fairly comprehensive picture of the positivity and negativity of emotions. Affective components were rated as more divergent than cognitive components. Cross-culturally, Americans and Canadians gave higher valence ratings to the salient valence of each emotion, and lower ratings to the non-salient valence of an emotion, compared to Chinese and Koreans. The results suggest that emotions encompass both positivity and negativity, and there were cross-cultural differences in reported emotions. This paradigm complements existing emotion theories, building on past research and allowing for more parsimonious explanations of cross-cultural research on emotion.

## Introduction

Emotion is widely studied and investigated in psychological science. It is a construct important for art, literature, and everyday life, and is at the core of the human experience. Traditionally, emotions are categorized dichotomously, as negative or positive, unpleasant or pleasant, activated or deactivated, and so on (Plutchik, 1980; Russell, 1980). Dichotomous classifications, however, are difficult to reconcile with certain developments in emotion research. In this paper, rather than classifying emotions on a single continuum from positive to negative, we argue that each emotion contains some degree of both negativity and positivity. Further, we employ a cross-cultural paradigm in an effort to highlight the nuances of the positivity and negativity of emotion, as there are several important differences in the affective signature of emotions across cultures. If this conceptualization is supported, it would facilitate more parsimonious explanations of several findings related to emotion. We investigated the issue with six basic emotions (sadness, fear, disgust, anger, surprise, and happiness; Ekman, 1992) in a cross-cultural study, involving participants from four different countries (South Korea, China, Canada, and the US).

### A Brief History of Emotion Classification

Emotions have long been an important topic of human interactions and society, and have hence been extensively studied in the field of psychological science. Perhaps the first notable scientific endeavor on emotions was Darwin’s (1872/1998). Darwin’s (1872/1998) concept of emotion as expressive and as physiological states has strongly influenced subsequent investigations of emotion. Consequently, emotion researchers have emphasized physiological aspects of emotions, particularly facial expressions (Ekman, 1977, 1999). Ekman’s research suggests that there are universal emotions based on cross-cultural recognition of facial expressions. Although there are cultural differences in display rules for expressing emotion, six basic emotions (sadness, fear, disgust, anger, surprise, and happiness) have been identified as universal. This tenet is widely accepted among emotion researchers.

In another early attempt to understand emotion, Wundt (1897) proposed that all emotions can be described by three dimensions: pleasurable to non-pleasurable, arousing to subduing, and strained to relaxed. Later, Schlosberg (1954) refined these three dimensions into pleasantness–unpleasantness, attention–rejection, and level of activation. Dimensional models of emotion are based on the assumption that all emotions involve the same interconnected system for emotion states. Still, the currently dominant models are the two-dimensional models: the circumplex model (Russell, 1980; Plutchik and Conte, 1997; Remington et al., 2000), the vector model (Bradley et al., 1992), and the Positive Activation – Negative Activation (PANA) model which contains two dimensions: positive to negative valence, and high to low activation (Watson and Tellegen, 1985). In most (if not all) of these models, valence is one important dimension, along which a specific emotion is categorized as positive or negative. Indeed, the field of scientific studies on emotion has rested and evolved on the assumption that emotions are either positive or negative.

Our research is motivated by the fact that certain developments in emotion research, including findings related to simultaneous feelings of positive and negative emotion and emotions having both positive and negative consequences, are difficult to explain by traditional, dichotomous classifications of emotion, which would require numerous auxiliary assumptions to satisfy. In the review below, we first briefly review these two areas generally, and then as they relate to culture. Next we review research related to the cognitive and affective components of emotion, and finally research on culture and dialectical thinking in order to frame our hypotheses.

### Positivity and Negativity in Emotion Research

First, research suggests that there are simultaneous positive and negative outcomes associated with specific emotions. Emotions can also have conflicting consequences; a positive emotion can lead to a negative consequence, and a negative emotion can lead to a positive consequence. For example, although happiness is considered as a positive and desirable emotion, research has revealed a darker side of happiness. According to Gruber et al. (2011), the pursuit of happiness is not always positive, and experiencing happiness is not always a good thing. People who pursue happiness strongly tend to be more depressed, miserable, and unhappy.

Further, participants primed with happy emotions displayed significantly more selfishness than those primed with sad emotions (Tan and Forgas, 2010). People placing a high importance on pursuing happiness reported significantly more loneliness compared to those neutral in pursuing happiness. Further, Tamir and Bigman (2014) demonstrate that there are benefits (cognitive upsides; e.g., anger resulted in better performance on a confrontational task) to experiencing emotions that typically elicit unpleasant feelings (affective downsides). This suggests that so-called positive emotions can have a negative influence on people, and that negative emotions can have a positive influence.

Second, regarding simultaneous positive and negative emotions, people reported simultaneously feeling happy and sad in various situations (Larsen et al., 2001; e.g., on their graduation day or the day moving away from home), despite that sadness and happiness were rated as opposites. Simultaneous emotions have also been reported in response to winning in disappointing fashion (e.g., a victory that could have been better) and losing in a relieving fashion (e.g., a defeat that could have been worse; Larsen et al., 2004). Thus, despite the traditional emotion framework that conceptualizes emotions dichotomously – as being either positive or negative, the literature suggests that a more flexible approach is needed. This might be why emotion researchers have long argued that the same emotion can serve different functions (Ekman, 1984; Scherer, 1984; Frijda and Mesquita, 1994). Indeed, researchers have found both positive and negative effects of a positive mood, and of a negative mood (Clore et al., 1994).

Bagozzi et al. (1999) investigated the valence of emotion cross-culturally, and found that Easterners tended to report more concurrently reported positive and negative emotions than Westerners. Further, Miyamoto et al. (2010) investigated the co-occurrence of positive and negative emotions in both Japan and the United States. Japanese participants reported feeling more mixed emotions than Americans in predominantly positive situations, but not in predominantly negative situations, in which there were no differences.

Cross-cultural research casts doubt on the classification of emotions as strictly positive or negative, because an emotion that is considered negative in one culture can be considered positive in another (Eid and Diener, 2001). Individualistic cultures believe that self-reflective emotions (emotions that reflect on the individual’s own actions) concerning a person doing well are good, whereas collectivistic cultures believe that self-reflective emotions concerning that one’s actions need improvement are desirable. For example, those from individualistic cultures tend to consider pride as a desirable emotion, and those from collectivist cultures tend to consider guilt a desirable emotion (Eid and Diener, 2001). This is in line with cultural differences in regulatory focus. Overall, Eid and Diener’s (2001) results further support that not all positive emotions are interpreted positively by all people, nor are all negative emotions interpreted negatively. Indeed, Tsai et al. (2006) and Tsai (2007) have shown that ideal affect – the affective state that people desire feeling – varies across cultures: Americans value high-arousal positive affective states (such as excitement) more, whereas East Asians value low-arousal positive affective states (such as calm or peacefulness) more. Further insight to these differences can be found in the literature concerning the components of emotion.

### Emotional Components

Research has indicated that emotion has affective and cognitive components (Scherer, 1982, 1984; Aue et al., 2007; Aue and Scherer, 2008; Dan Glauser and Scherer, 2008; Grandjean and Scherer, 2008). The affective component is related to our immediate response to stimuli (feelings), while the cognitive component is our more controlled, conscious evaluation after the immediate response (thinking). The Component Process Model (CPM), which is based on appraisal theory, suggests that emotion is composed of different stages: automatic sensory motor, schematic unconscious, and controlled conscious stages (Scherer, 1982, 1984; Van Reekum and Scherer, 1997). According to the CPM, the affective process is more immediate and provides a strong subjective sense of feeling that is prominent to oneself. The affective process is automatic, less controlled, and tends to be strongly aligned with the valence of the emotion); on the other hand, the cognitive process is rather controlled, occurring later in the realization of emotion sequence. The cognitive components of emotions are therefore considered less intense and less extreme compared to the affective components (Scherer, 1982, 1984; Tice et al., 2004; Ochsner and Gross, 2005). Indeed, research has shown that affective and cognitive components of emotions do not necessarily corroborate each other. For example, Tamir and Bigman (2014) explain that emotions can be pursued both for how they feel and what they accomplish, and highlight the complexities among the different emotional motives. For example, a boxer may desire to feel anger, a negative emotion, in order to motivate himself to beat his opponent. So although he may be feeling a negative emotion (affective component), he thinks about it in a positive light (cognitive component).

This distinction is similar to Mayer and Gaschke’s (1988) model, which argues that moods have at least two components: The direct experience of a mood, and a meta level of experience that consists of thoughts and feelings about the mood (Mayer and Gaschke, 1988). They are called meta-moods because they do not concern the immediate experience of feeling states, but concern their reflective experience, involving subjective thoughts and feelings about one’s moods. The regulatory process of the reflective experience is under the individual’s control. The duality described above may explain why some cultures focus on hedonic pleasures (e.g., Western cultures) while others focus on peace and harmony (e.g., Eastern cultures). Indeed, East Asians reported that happiness is experienced as rather calm and zen-like, whereas Westerners reported that happiness is experienced as excitement and extreme positivity (Tsai, 2007). This may explain why Westerners desire to feel positive emotions more than negative emotions relative to Easterners (Sims et al., 2015). All of the above suggests that subjective interpretation of specific emotions (i.e., primarily the cognitive component of emotion) differs across cultures (Uchida et al., 2004). Most recently, an argument based on Higher Order Theory (HOT) was put forth that emotions are cognitive (LeDoux and Brown, 2017). Based on the literature review on neuroimaging evidence, they have concluded that emotion is a cognitive process, as it involves cortical (conscious) rather than subcortical (unconscious) brain circuits. This suggests that emotion could be a higher order process, separate from the affect we feel.

Emotions and cognitions are intertwined. Indeed, emotions are the result of human cognition (Lazarus, 1982), and cognitive processes in turn are influenced by emotion (Forgas, 1995). Research has shown that culture influences cognitive processes (Nisbett et al., 2001). Ji et al. (2001) found that Westerners tend to have a linear thinking style, resulting in expectations of unchanging or consistent outcomes. Easterners, by contrast, have a more dialectical thinking style, resulting in expectations of more malleable or changing outcomes (see also Peng and Nisbett, 1997). East Asians’ greater dialectical thinking, or the degree to which contradictions are tolerated, is thought to drive the more predominant emotional complexity (i.e., concurrence of positive and negative emotion) of East-Asians relative to Westerners (Spencer-Rodgers et al., 2010). Because dialectical thinking involves a greater expectation of change, it is likely that any thought about emotion will be less extreme. This is because if something is expected to change or fluctuate, it would not be taken in the extreme view. Linear thinkers, on the other hand, expect stability, so their thoughts might be grounded more in the extremes (less likely to change because they are so strong). One difference between dialectical and linear thinking styles lies in how people from different cultures view events. Easterners tend to take a more balanced view of negative events compared to Westerners (Ji et al., 2004). Indeed, findings suggest that people from dialectical and collectivist cultures (Chinese, Koreans, Japanese, Indonesians, and Malaysians) make less polarized life quality judgments, whether negative or positive, compared to non-dialectical thinkers and individualist cultures (e.g., Americans; Minkov, 2009).

Greater dialectical thinking among East Asians than European North Americans also contributes to cultural differences in emotion regulation. Miyamoto and Ma (2011), in a series of studies, showed that, although people in general want to savor rather than dampen their positive emotions, such hedonic emotion regulation was weaker among Asians than among Americans. Furthermore, dialectical beliefs about positive emotions mediated such cultural differences. As a whole, these findings indicate that culturally specific dialectical beliefs guide both emotion regulation and emotional experience. In fact, people from interdependent cultures tend to report experiencing positive and negative emotions simultaneously (Spencer-Rodgers et al., 2010). This suggests that dialecticism results in emotional complexity.

There are individual and cultural variations in how positive and negative emotions are perceived (Eid and Diener, 2001; Miyamoto et al., 2014; Sims et al., 2015). For instance, Luong et al. (2016) reported evidence that people differ in negative affect valuation – the extent to which negative affective states are valued as pleasant, useful/helpful, appropriate, and meaningful experience, which has implications for the links between affective experience and psychological/physical well-being. Furthermore, Riediger et al. (2014) showed evidence through experience sampling that people experience both positive and negative (i.e., mixed) affects simultaneously, which was especially true for adolescents and younger adults. Cross-culturally, Miyamoto et al. (2014) found that Asians were less likely than Euro-Americans to engage in hedonic emotion regulation after experiencing a negative event or feeling a negative emotion, mediated by stronger dialectical beliefs among Asians than among Euro-Americans. Indeed, Asians had a more positive view of negative emotions, as they were more likely than Euro-Americans to believe in the motivational and cognitive utility of negative emotions.

One issue is that although previous research suggests the possibility that each emotion contains both positivity and negativity, most researchers have relied on bipolar measures to assess the cognitive and affective components of emotions, which made it impossible to examine the extent to which different emotions are ambivalent, containing both positivity and negativity. The present research extends the earlier work by taking a new approach in methodology, in which we measure positivity and negativity of emotion separately.

## Hypotheses

Based on the theoretical and empirical evidence reviewed above, we generated three hypotheses. First, although most emotions may be most salient in either positivity (e.g., in the case of happiness) or negativity (e.g., in the case of sadness), we hypothesized that each emotion would contain some degree of both positivity and negativity (H1). Second, based on research that the affective component of emotion is more intense and extreme than the cognitive component, we hypothesized that the positivity and negativity ratings would be more divergent for affective than for cognitive components. For example, sadness, which is salient in negativity, should be rated more negatively on the affective than the cognitive component, and relatively less positively on the affective than cognitive component (H2). Finally, based on the review of the culture and emotion literature, we hypothesized that American and Canadian ratings would be higher for the salient valence (e.g., negativity for sadness) and lower for the less salient valence (e.g., positivity for sadness) of an emotion, compared to Chinese and Koreans (H3).

## Materials and Methods

### Participants

Our sample included a total of 458 undergraduate students (166 men and 292 women) recruited in four countries: 147 South Koreans (61 men and 86 women, mean age 20.51, *SD* = 2.04), 90 Chinese (44 men and 46 women, mean age 18.87, *SD* = 1.06), 109 Euro-Canadians (15 men and 94 women, mean age 19.11, *SD* = 6.96), and 112 Americans (46 men and 66 women, mean age 21.39, *SD* = 5.76). The Americans were Caucasians (30%), Hispanics (62%), and others (8%; Native Americans, Black, etc.); however, Asians were excluded from the American sample. Preliminary analyses on ethnicity in the American sample revealed no significant differences between Caucasians and Hispanics (*ps* ≥ 0.263). All participants participated in exchange for partial course credit or small gifts. Participants from South Korea and China were all nationally and ethnically Koreans and Chinese, respectively. Participants from Canada and the US were all nationally Canadians and Americans, respectively.

### Design and Procedure

The experiment employed a 6 (emotions: sadness, fear, disgust, anger, happiness, and surprise) × 2 (valence: negative vs. positive) × 2 (emotional component: affective vs. cognitive) × 4 (culture: South Korea, China, Canada, US) mixed participants design, with the first three variables as within-participant factors. In order to avoid culturally specific or unique emotions, we employed the six basic emotions, as research suggests that these emotions are universal (Ekman, 1992).

The study was conducted in a lab setting, with all measures presented on a computer monitor. First, participants thought about each basic emotion (presented in a random order), and then responded to two questions measuring the affective positivity and negativity, and cognitive positivity and negativity of each emotion in a random order, respectively, on a scale from 0 (not at all) to 6 (extremely). Take sadness as an example: We asked two questions to measure sadness’s affective positivity (“*How positive does experiencing sadness feel to you?*” “*How good do you feel when you are feeling sad?*”), affective negativity (“*How negative does experiencing sadness feel to you?*” “*How bad do you feel when you are feeling sad?*”), cognitive positivity (“*To what extent do you think the emotion of sadness is positive?*” “*Overall, how positive do you think experiencing the emotion of sadness is?*”), and cognitive negativity (“*To what extent do you think the emotion of sadness is negative?*” “*Overall, how negative do you think experiencing the emotion of sadness is*?”).

The study was conducted in the participants’ native language. Study materials were developed in English, and then translated into Chinese and Korean for those respective samples. Several bilingual research assistants assisted with the translation, or checked the back translation for equivalence across languages. The first two authors also reviewed the translation to ensure equivalence across languages. See **Table 1** for the Korean, Chinese, and English translations of each emotion.

**Table 1:** Six emotions in Korean, Chinese, and English.

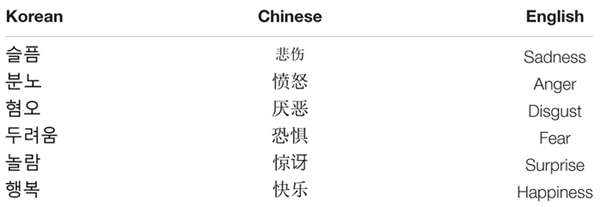

## Results

### Item Reliability

Given that we used two items to measure each valence (negativity and positivity) for each emotion (sadness, fear, disgust, anger, surprise, and happiness), for the affective and cognitive components, respectively, we first calculated the correlations between these two items before averaging them. The correlations ranged from 0.59 to 0.73 across emotions for affective positivity ratings, and from 0.46 to 0.76 for affective negativity ratings, *ps* < 0.001. The correlations ranged from 0.58 to 0.78 for cognitive positivity ratings, and from 0.65 to 0.80 for cognitive negativity ratings, *ps* < 0.001. Thus, the two items showed reasonable reliability overall, so we used the mean of the two items in subsequent ANOVA analyses. A preliminary analysis revealed no significant main effects or interactions involving gender, and therefore gender is not included in the following analyses.

### The Coexistence of Positivity and Negativity

**FIGURE 1:**
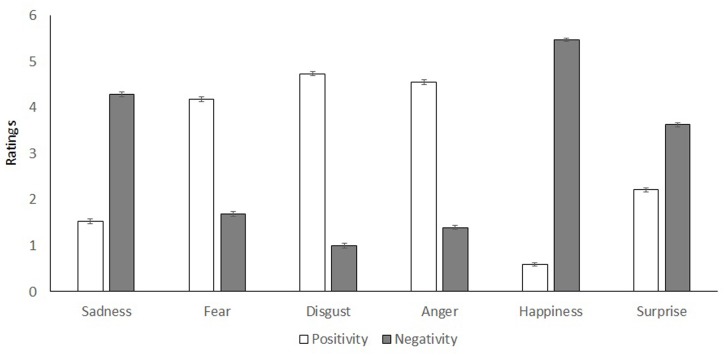
**Comparison of the positivity and negativity of emotions (0 = Not at all; 6 = Extremely)**.

**FIGURE 2:**
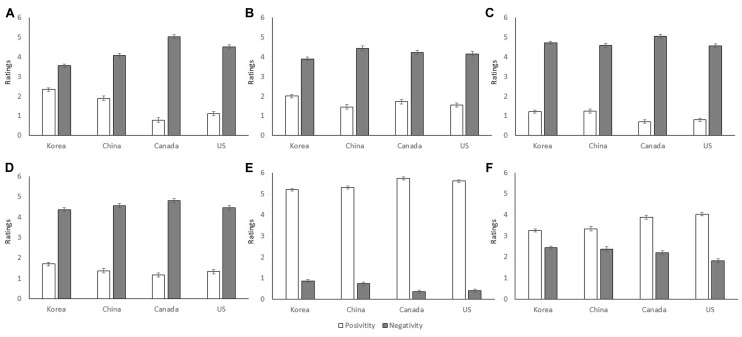
**Positivity and negativity of emotions across countries. (A)** Sadness, **(B)** Fear, **(C)** Disgust, **(D)** Anger, **(E)** Happiness, and **(F)** Surprise. (0 = Not at all; 6 = Extremely).

To test Hypothesis 1, we compared the average positivity and negativity ratings of the affective and cognitive components for each emotion to zero (which means “not at all” positive or negative) and found all the comparisons significant, *ts* > 13.58, *ps* < 0.001. Thus, H1, that each emotion contained some degree of positivity and negativity is supported (**Figure 1**). Further analyses showed that the ratings of positivity and negativity ratings were most divergent for happiness, followed by disgust, anger, sadness, fear, and surprise, in that order, *Fs*(1,457) ≥ 4.20, *ps* ≤ 0.041.^1^ If less divergent ratings of positivity and negativity indicate ambivalence, the results showed that surprise is a much more ambivalent emotion than happiness, with anger, disgust, fear and sadness in between. Such between-emotion differences cannot be explained by response biases, such as random responding or acquiescence. Additionally, we performed a 6 (emotion) × 2 (valence) × 4 (country) mixed ANOVA with emotional ratings averaged across affective and cognitive components as the dependent variable. There was a significant main effect of emotion, *F*(5,454) = 14.10, *p* < 0.001, $\eta_{p}^{2}$ = 0.03; this was qualified by a two-way interaction between emotion and valence, *F*(5,454) = 1821.10, *p* < 0.001, $\eta_{p}^{2}$ = 0.80, a two-way interaction between emotion and country, *F*(15,454) = 4.77, *p* < 0.001, $\eta_{p}^{2}$ = 0.03, and a three-way interaction between emotion, valence, and country, *F*(15,454) = 22.68, *p* < 0.001, $\eta_{p}^{2}$ = 0.13 (see detailed results in **Figure 2** and subsequent analyses in the following section). Further analyses showed that sadness, fear, surprise and happiness were more ambivalent to Chinese/Koreans than to Canadians/Americans, *Fs* ≥ 3.65, *ps* ≤ 0.013. Again, these between-emotional and cross-cultural differences cannot be explained by random responding. See **Table 2** for mean differences of each emotion across countries.

**Table 2:** Valences and components of six emotions in Korea, China, Canada, and the US.

			Sadness		Fear		Disgust		Anger		Happiness		Surprise	
			*M*	*SD*	*M*	*SD*	*M*	*SD*	*M*	*SD*	*M*	*SD*	*M*	*SD*
Korea	Negative	Affective	3.80	1.25	4.03	1.18	4.83	1.21	4.64	1.20	0.83	1.09	2.42	1.11
		Cognitive	3.30	1.38	3.77	1.38	4.61	1.31	4.11	1.36	0.89	1.02	2.45	1.20
	Positive	Affective	1.87	1.30	1.76	1.38	1.01	1.17	1.34	1.28	5.20	1.05	3.17	1.11
		Cognitive	2.81	1.99	2.27	1.39	1.44	1.33	2.07	1.48	5.21	0.93	3.35	1.15
China	Negative	Affective	4.28	1.18	4.62	1.09	4.71	1.15	4.75	1.12	0.66	0.83	2.41	1.11
		Cognitive	3.86	1.34	4.28	1.27	4.46	1.17	4.38	1.19	0.81	0.90	2.36	1.07
	Positive	Affective	1.66	1.22	1.24	1.06	1.06	0.94	1.12	0.98	5.34	0.75	3.29	0.95
		Cognitive	2.11	1.35	1.69	1.16	1.46	1.17	1.63	1.24	5.27	0.82	3.38	0.97
Canada	Negative	Affective	5.28	0.81	4.39	1.26	5.02	0.91	4.87	1.00	0.39	0.77	2.13	1.17
		Cognitive	4.78	1.18	4.07	1.17	5.09	0.98	4.74	0.99	0.34	0.64	2.29	1.18
	Positive	Affective	0.49	0.59	1.46	1.18	0.67	0.77	0.98	0.93	5.70	0.52	3.81	1.16
		Cognitive	1.09	1.08	1.99	1.28	0.75	0.95	1.36	1.08	5.78	0.47	3.94	1.14
US	Negative	Affective	4.67	1.18	4.21	1.33	4.42	1.31	4.43	1.27	0.29	0.54	1.71	1.17
		Cognitive	4.37	1.39	4.12	1.46	4.73	1.12	4.50	1.21	0.54	1.02	1.92	1.20
	Positive	Affective	0.75	0.89	1.22	1.34	0.68	0.92	1.20	1.17	5.64	0.68	4.00	1.03
		Cognitive	1.50	1.46	1.88	1.52	0.93	1.10	1.47	1.25	5.58	0.92	4.08	1.06

*N_Korea_ = 147, N_China_ = 90, N_Canada_ = 109, and N_US_ = 112.*

Finally, we used the MIN statistic (Schimmack, 2001) to test H1. According to Schimmack, the intensity of the weaker rating in valence, is a “more appropriate statistic (p92)” to test whether positivity and negativity are mutually exclusive. If positivity and negativity belonged to the same dimension (as unipolar opposites), then MIN value should be close to zero. We computed MIN value as the lower of the two valence ratings (i.e., I[V] = MIN[I[P],I[N]]), with I[V] being the MIN score, I[P] the ratings of positivity and I[N] the ratings of negativity. Comparing this MIN score to zero within each emotion and each culture, we found that all MIN scores were greater than zero (*ps* < 0.001), supporting H1.

We then conducted a 4 (country) × 6 (emotion) mixed ANOVA on MIN scores. There was a significant main effect of country, *F*(3,454) = 18.62, *p* < 0.001, $\eta_{p}^{2}$ = 0.11. *Post hoc* analyses showed that Koreans and Chinese were not different from each other (*p* = 0.111), Canadians and Americans were not different from each other (*p* = 0.826), but the two East Asian groups significantly differently from the two North American groups (*ps* < 0.001). There was a significant main effect of emotion, *F*(5,452) = 222.19, *p* < 0.001, $\eta_{p}^{2}$ = 0.71. *Post hoc* analyses showed that the MIN scores for anger and sadness did not differ from each other (*p* = 0.201), but all other pair-wise comparisons between any two emotions were significant (*ps* < 0.001). In addition, these main effects were qualified by a significant two-way interaction between country and emotion, *F*(24,434) = 25.19, *p* < 0.001, $\eta_{p}^{2}$ = 0.22. See **Table 3**.

**Table 3:** MIN Scores of Six Emotions in Korea, China, Canada, and the US.

	Sadness		Fear		Disgust		Anger		Happiness		Surprise	
	*M*	*SD*	*M*	*SD*	*M*	*SD*	*M*	*SD*	*M*	*SD*	*M*	*SD*
Korea	1.97	0.99	1.74	0.92	1.16	1.04	1.56	1.07	0.83	0.89	2.14	0.86
China	1.70	0.97	1.44	0.93	1.23	0.90	1.30	0.85	0.71	0.73	2.24	0.89
Canada	0.79	0.71	1.62	0.88	0.71	0.75	1.17	0.88	0.36	0.57	2.03	0.99
US	1.05	0.92	1.32	1.01	0.78	0.84	1.26	0.98	0.42	0.66	1.75	0.94

*N_Korea_ = 147, N_China_ = 90, N_Canada_ = 109, and N_US_ = 112.*

We examined each emotion separately to better understand the interaction effects. Simple effect analyses showed that for anger, Koreans had a higher MIN score than the other three groups (*ps* ≤ 0.042)^2^ while the other three groups did not differ from one another (*ps* > 0.352). For sadness, each country was significantly differently from any other country (*ps* ≤ 0.038)^3^. For fear, Koreans were significantly higher than Chinese and Americans (*ps* ≤ 0.015) and Canadians were significantly higher than Americans (*p* = 0.017), but no other pair-wise comparisons were significant (*ps* ≥ 0.166). For disgust, Koreans and Chinese were significantly higher than Canadians or Americans (*ps* ≤ 0.001), with no significant difference between Koreans and Chinese or between Canadians and Americans (*ps* ≥ 0.538). For happiness, Koreans and Chinese were significantly higher than Canadians or Americans (*ps* ≥ 0.005), with no significant difference between Koreans and Chinese or between Canadians and Americans (*ps* ≤ 0.234). For surprise, Americans were significantly lower than each of the other groups (*ps* ≤ 0.025)^4^, whereas the other three groups did not differ from one another (*ps* ≥ 0.094). Given that all MIN scores were significantly greater than zero, we conclude that positivity and negativity are not mutually exclusive, and can co-exist in each emotion. Differences in each emotion are addressed in the following analyses of H2 and H3.

Before further hypothesis testing, we conducted a series of exploratory factor analyses (EFA) in order to confirm that participants’ cognitive and affective component responses actually loaded on separate factors. First, we ran a principal axis factor analysis with varimax rotation on each of the six emotions. For each emotion except surprise, examination of the rotated factor matrices revealed the best fit was a two-factor solution with the cognitive components on one factor and the affective components on the other, and within each factor, the positive and negative component loading on opposite sides (i.e., positive factor loadings were positive, and negative factor loadings were negative). For surprise, the rotated factor matrix revealed positive components (both affective and cognitive) loaded on one factor, and negative components (both affective and cognitive) on the other.

In order to confirm the results of the EFAs, confirmatory factor analyses (CFA) using Maximum Likelihood Estimation were conducted using AMOS software. For each emotion, both one-factor (cognitive and affective together) and two-factor (cognitive and affective separately) models were run. For all six emotions, the two-factor model was a better fit than the one-factor model based on several fit indices (GFI, CFI, and RMSEA). See **Table 4**. Taken together, the analyses support the separation of the cognitive and affective components.

**Table 4:** Goodness-of-fit indicators of models of six emotions.

		Fit index		
Emotion	Model	GFI	CFI	RMSEA
Sadness	Single factor	0.72	0.74	0.28
	Two factor	0.78	0.83	0.24
Fear	Single factor	0.74	0.72	0.27
	Two factor	0.86	0.85	0.20
Disgust	Single factor	0.74	0.73	0.27
	Two factor	0.79	0.82	0.23
Anger	Single factor	0.77	0.77	0.24
	Two factor	0.87	0.88	0.18
Happiness	Single factor	0.79	0.77	0.22
	Two factor	0.88	0.88	0.16
Surprise	Single factor	0.65	0.63	0.32
	Two factor	0.74	0.78	0.25

*N = 458.*

To examine H2 and H3, we conducted a 2 (valence: negative vs. positive) × 2 (emotional component: affective vs. cognitive) × 6 (emotion) × 4 (culture: South Korea, China, Canada, and the US) mixed ANOVA, with the former three variables as within-participant factors. (Due to the large number of analyses involved, we report here only those analyses most relevant to testing our hypotheses). Overall, participants rated the emotions as more negative (*M* = 3.43, *se* = 0.03) than positive (*M* = 2.45, *se* = 0.03), *F*(1,454) = 370.25, *p <* 0.001, $\eta_{p}^{2}$ = 0.45. This likely reflects that fact that the majority in the basic emotions are more negative than positive (e.g., sadness, fear, disgust, and anger). Regarding H2 that the positivity and negativity ratings would be more divergent for affective than for cognitive components, the Valence × Component interaction was significant, *F*(1,454) = 97.09, *p <* 0.001, $\eta_{p}^{2}$ = 0.18. Specifically, participants in general rated the emotions as more negative on the affective component (*M* = 3.49, *se* = 0.03) than on the cognitive component (*M* = 3.37, *se* = 0.03), *F*(1,454) = 162.24, *p <* 0.001, $\eta_{p}^{2}$ = 0.26, but positive on the cognitive component (*M* = 2.63, *se* = 0.04) than on the affective component (*M* = 2.28, *se* = 0.03), *F*(1,454) = 21.65, *p <* 0.001, $\eta_{p}^{2}$ = 0.05. This provides support to H2. Due to higher level interactions, a Valence × Component × Culture interaction, *F*(3,454) = 4.42, *p* = 0.004, $\eta_{p}^{2}$ = 0.03, a Valence × Component × Emotion interaction, *F*(5,452) = 23.13, *p* < 0.001, $\eta_{p}^{2}$ = 0.20, and a Valence × Component × Emotion × Culture interaction, *F*(15,1356) = 1.99, *p* = 0.013, $\eta_{p}^{2}$ = 0.02, we will examine H2 more closely within each emotion.

In regard to H3 that American and Canadian ratings would be higher for the salient valence (e.g., negativity for sadness) and lower for the less salient valence (e.g., positivity for sadness) of an emotion, compared to Chinese and Koreans, the valence × emotion × country interaction was significant, *F*(15,1356) = 16.38, *p <* 0.001, $\eta_{p}^{2}$ = 0.15. In addition, the Valence × Component × Emotion × Culture interaction was significant, *F*(15,1356) = 1.99, *p* = 0.013, $\eta_{p}^{2}$ = 0.02. To better understand these interactions, we examined each emotion separately.

### Sadness

To test H2 that the positivity and negativity ratings would be more divergent for affective than for cognitive components, we conducted a 2 (valence: negativity vs. positivity) × 2 (component: cognitive vs. affective) × 4 (country) mixed ANOVA on the average ratings. The most relevant result to test H2 is the two-way interaction between valence and component, *F*(1,454) = 110.84, *p* < 0.001, $\eta_{p}^{2}$ = 0.20, which was not qualified by a three-way interaction, *F*(3,454) = 1.40, *p* = 0.243. Simple effect analyses revealed that, consistent with H2, sadness was rated more negatively on the affective component (*M*_affective & negativity_ = 4.51, *se* = 0.05) than on the cognitive component (*M*_cognitive & negativity_ = 4.08, *se* = 0.06), but more positively on the cognitive component (*M*_cognitive_ _&positivity_ = 1.88, *se* = 0.07) than on the affective component (*M*_affective & positivity_ = 1.19, *se* = 0.05), *ts* > 7.60, *ps* < 0.001.

Regarding H3 that Americans and Canadians would rate sadness as more negative and less positive than Chinese and Koreans, there was a significant two-way interaction between country and valence, *F*(3,454) = 54.33, *p* < 0.001, $\eta_{p}^{2}$ = 0.26, which was not qualified by a three-way interaction, *F*(3,454) = 1.40, *p* = 0.243. Simple effect analyses revealed that Canadians (*M*_Canada & negativity_ = 5.03, *se* = 0.10) and Americans (*M*_US & negativity_ = 4.52, *se* = 0.10) rated sadness to be more negative than Chinese (*M*_China & negativity_ = 4.07, *se* = 0.11), and Koreans (*M*_Korea & negativity_ = 3.55, *se* = 0.09), *Fs* > 43.42, *ps* < 0.017, whereas Koreans (*M*_Korea & positivity_ = 2.34, *se* = 0.10) and Chinese (*M*_China & positivity_ = 1.89, *se* = 0.12) rated sadness to be more positive than Americans (*M*_US & positivity_ = 1.12, *se* = 0.11) and Canadians (*M*_Canada & positivity_ = 0.79, *se* = 0.11), *Fs* > 46.61, *ps* < 0.019 Thus, both H2 and H3 were fully supported.

### Fear

A 2 (valence: negativity vs. positivity) × 2 (component: cognitive vs. affective) × 4 (country) mixed ANOVA on the average ratings of fear revealed a significant interaction between valence and component, *F*(1,454) = 56.67, *p* < 0.001, $\eta_{p}^{2}$ = 0.11, which was not qualified by a three-way interaction, *F*(3,454) = 0.04, *p* = 0.99. Simple effect analyses revealed that, consistent with H2, fear was rated more negatively on the affective component (*M*_affective & negativity_ = 4.31, *se* = 0.06) than on the cognitive component (*M*_cognitive & negativity_ = 4.06, *se* = 0.06), but more positively on the cognitive component (*M*_cognitive_ _&positivity_ = 1.95, *se* = 0.06) than on the affective component (*M*_affective & positivity_ = 1.42, *se* = 0.06), *ts* > 4.38, *ps* < 0.001. Thus, H2 was fully supported.

In addition, there was a significant two-way interaction between country and valence, *F*(3,454) = 5.43, *p* < 0.001, $\eta_{p}^{2}$ = 0.04. Simple effect analyses revealed that Chinese (*M*_China & negativity_ = 4.45, *se* = 0.12), Canadians (*M*_Canada & negativity_ = 4.23, *se* = 0.11) and Americans (*M*_US & negativity_ = 4.16, *se* = 0.11) rated fear to be more negative compared to Koreans (*M*_Korea & negativity_ = 3.90, *se* = 0.09), although only the Koreans vs. Chinese comparison was significant, *F*(3,454) = 4.71; *p* = 0.003; for all the other comparisons, *Fs* < 4.71, *ps* > 0.125. Meanwhile Koreans (*M*_Korea & positivity_ = 2.01, *se* = 0.09) and Canadians (*M*_Canada & positivity_ = 1.72, *se* = 0.11) rated fear to be more positive than Americans (*M*_US & positivity_ = 1.55, *se* = 0.11) and Chinese (*M*_China & positivity_ = 1.46, *se* = 0.12), although only comparisons involving Koreans vs. Chinese and Koreans vs. Americans were significant, *Fs* > 5.52; *ps* < 0.001). Thus, H3 was only partially supported.

### Disgust

A 2 (valence: negativity vs. positivity) × 2 (component: cognitive vs. affective) × 4 (country) mixed ANOVA on the average ratings of disgust revealed a significant interaction between valence and component, *F*(3,454) = 13.00, *p* < 0.001, $\eta_{p}^{2}$ = 0.03, which was qualified by a three way interaction among valence, component, and country, *F*(3,454) = 5.29, *p* < 0.001, $\eta_{p}^{2}$ = 0.03. Thus we decided to investigate H2 within each country.

The 2 (valence) × 2 (component) interaction was significant for Koreans, *F*(1,146) = 18.31, *p* < 0.001, $\eta_{p}^{2}$ = 0.11, and Chinese, *F*(1,89) = 9.25, *p* = 0.003, $\eta_{p}^{2}$ = 0.09, but not for Canadians, *F*(1,108) = 0.00, *p* = 0.976, $\eta_{p}^{2}$ = 0.00, nor Americans, *F*(1,111) = 11, *p* = 0.739, $\eta_{p}^{2}$ = 0.00. The simple effect analyses revealed that Koreans rated disgust more negatively on the affective (*M*_Korea & affective & negativity_ = 4.83, *se* = 0.10) than cognitive component (*M*_Korea & cognitive & negativity_ = 4.61, *se* = 0.10), and more positively on the cognitive (*M*_Korea & cognitive_ _&positivity_ = 1.44, *se* = 0.10) than affective component (*M*_Korea & affective & positivity_ = 1.01, *se* = 0.08), *ts* > 2.53, *ps* < 0.012. Next, the simple effect analyses revealed that Chinese rated disgust more negatively on the affective (*M*_China & affective & negativity_ = 4.71, *se* = 0.12) than the cognitive component (*M*_China & cognitive & negativity_ = 4.46, *se* = 0.12), *t* = −3.74, *p* < 0.001, and more positively on the cognitive (*M*_China & cognitive & positivity_ = 1.46, *se* = 0.12) than the affective component (*M*_China & affective & positivity_ = 1.06, *se* = 0.10), *t* = 1.98, *p* = 0.051. Thus, H2 was supported for Korean and Chinese participants, but not for Canadian or American participants.

Due to the significant 3-way interaction, we examined H3 within affective and cognitive components separately. Within the affective component, the simple effect analyses revealed that Canadians (*M*_Canada & affective & negativity_ = 5.02, *se* = 0.11) rated strongest affective negativity for disgust followed by Koreans (*M*_Korea & affective & negativity_ = 4.83, *se* = 0.10), Chinese (*M*_China & affective & negativity_ = 4.71, *se* = 0.12), and Americans (*M*_US & affective & negativity_ = 4.42, *se* = 0.11); Canadians were significantly different from Chinese and Americans, and Koreans were significantly different from Americans, *Fs* > 5.23, *ps* < 0.030; none of the other comparisons were significant, *Fs* < 5.23, *ps* > 0.382. In addition, for affect positivity, the simple effect analyses revealed that Canadians (*M*_Canada & affective & positivity_ = 0.67, *se* = 0.09) and Americans (*M*_US & affective & positivity_ = 0.68, *se* = 0.09) rated disgust less positively than did Koreans (*M*_Korea & affective & positivity_ = 1.01, *se* = 0.08) and Chinese (*M*_China & affective & positivity_ = 1.06, *se* = 0.10), *Fs* > 5.03, *p* < 0.041 for the East vs. West comparisons. This latter part is consistent with H3.

Within the cognitive component, simple effect analyses revealed that Canadians (*M*_Canada & cognitive & negativity_ = 5.09, *se* = 0.11) rated disgust more negatively than did Koreans (*M*_Korea & cognitive & negativity_ = 4.61, *se* = 0.10) and Chinese (*M*_China & cognitive & negativity_ = 4.46, *se* = 0.12), *Fs* > 5.65, *ps* < 0.007. Americans (*M*_US & cognitive & negativity_ = 4.73, *se* = 0.11), also rated disgust more negatively than the two Asian groups, although the difference did not reach statistical significance; *ps* > 0.603. In addition, the simple effect analyses revealed that Canadians (*M*_Canada & cognitive & positivity_ = 0.75, *se* = 0.11) and Americans (*M*_US & cognitive & positivity_ = 0.93, *se* = 0.11) rated disgust less positively than did Koreans (*M*_Korea & cognitive & positivity_ = 1.44, *se* = 0.10), and Chinese (*M*_China & cognitive & positivity_ = 1.46, *se* = 0.12), *Fs* > 10.84, *ps* < 0.009. Thus, H3 was partially supported in the cognitive component.

### Anger

A mixed ANOVA on the average ratings of anger revealed a significant interaction between valence and component, *F*(1,454) = 57.73, *p* < 0.001, $\eta_{p}^{2}$ = 0.11, which was qualified by a three way interaction among valence, component, and country, *F*(3,454) = 6.78, *p* < 0.001, $\eta_{p}^{2}$ = 0.04. Thus we decided to investigate H2 within each country.

The 2 (valence) × 2 (component) interaction was significant for Koreans, *F*(1,146) = 46.84, *p* < 0.001, $\eta_{p}^{2}$ = 0.24, Chinese, *F*(1,89) = 20.69, *p* < 0.001, $\eta_{p}^{2}$ = 0.19, and Canadians, *F*(1,108) = 8.39, *p* = 0.005, $\eta_{p}^{2}$ = 0.07, but not for Americans, *F*(1,111) = 1.30, *p* = 0.257, $\eta_{p}^{2}$ = 0.01. The simple effect analyses revealed that both Koreans and Chinese rated affective negativity (*M*_Korea & affective & negativity_ = 4.64, *se* = 0.10; *M*_China & affective & negativity_ = 4.75, *se* = 0.12) stronger compared to cognitive negativity (*M*_Korea & cognitive & negativity_ = 4.11, *se* = 0.10; *M*_China & cognitive & negativity_ = 4.38, *se* = 0.13), and cognitive positivity (*M*_Korea & cognitive & positivity_ = 2.07, *se* = 0.11; *M*_China & cognitive & positivity_ = 1.63, *se* = 0.14) stronger than affective positivity (*M*_Korea & affective & positivity_ = 1.34, *se* = 0.09; *M*_China & affective & positivity_ = 1.12, *se* = 0.12), *ts* > 3.54, *ps* < 0.001. Canadians showed the same pattern of results for negativity as Koreans and Chinese (*M*_Canada & affective & negativity_ = 4.87, *se* = 0.11; *M*_Canada & cognitive & negativity_ = 4.74, *se* = 0.12), *t* = −4.01, *p* < 0.001; however, they showed no significant differences between cognitive positivity (*M*_Canada & cognitive & positivity_ = 1.36, *se* = 0.12) and affective positivity (*M*_Canada & affective & positivity_ = 0.98, *se* = 0.11), *t* = 1.26, *p* = 0.210. Thus, H2 was fully supported in the Korean and Chinese samples, and partially supported in the Canadian sample.

Due to the significant 3-way interaction, we examined H3 within affective and cognitive components separately. Within the affective component, the simple effect analyses revealed that Canadians (*M*_Canada & affective & negativity_ = 4.87, *se* = 0.11) rated affective negativity the strongest, followed by Chinese (*M*_China & affective & negativity_ = 4.75, *se* = 0.12), Koreans (*M*_Korea & affective & negativity_ = 4.64, *se* = 0.10), and Americans (*M*_US & affective & negativity_ = 4.43, *se* = 0.11); only the difference between Americans and Canadians was significant, *F* = 2.83, *p* = 0.031. There were no significant differences among the four countries on affective positivity for anger (*M*_Canada & affective & positivity_ = 0.98, *se* = 0.11, *M*_China & affective & positivity_ = 1.12, *se* = 0.12, *M*_US & affective & positivity_ = 1.20, *se* = 0.11, *M*_Korea & affective & positivity_ = 1.34, *se* = 0.09), *F* = 2.23, *p* > 0.071.

Within the cognitive component, simple effect analyses revealed that Canadians (*M*_Canada & cognitive & negativity_ = 4.74, *se* = 0.12) and Americans (*M*_US & cognitive & negativity_ = 4.50, *se* = 0.11) rated anger more negatively than did Koreans (*M*_Korea & cognitive & negativity_ = 4.11, *se* = 0.10), *Fs* > 5.97, *p* < 0.001 and *p* = 0.057, respectively. Canadians and Americans also rated anger more negatively than did Chinese (*M*_China & cognitive & negativity_ = 4.38, *se* = 0.13), although these differences did not reach statistical significance; *ps* > 0.221. For cognitive positivity, Canadians (*M*_Canada & cognitive & positivity_ = 1.36, *se* = 0.12) rated anger least positively, followed by Americans (*M*_US & cognitive & positivity_ = 1.47, *se* = 0.12), Chinese (*M*_China & cognitive & positivity_ = 1.63, *se* = 0.14), and Koreans (*M*_Korea & cognitive & positivity_ = 2.07, *se* = 0.11), omnibus *F* = 7.71, *p* < 0.001, however, Chinese were not significantly different from Koreans, Canadians or Americans; *p* > 0.073. Thus, H3 was partially supported in the cognitive component when evaluating anger.

### Happiness

A mixed ANOVA revealed a marginally significant interaction between valence and component, *F*(1,454) = 2.79, *p* = 0.096, $\eta_{p}^{2}$ = 0.01, which was not qualified by a 3-way interaction, *F*(3,454) = 1.80, *p* = 0.147. Consistent with H2, happiness was rated more negatively on the cognitive component (*M*_cognitive & negativity_ = 0.54, *se* = 0.04) than the affective component (*M*_affective & negativity_ = 0.65, *se* = 0.04), *t* = −2.37, *p* = 0.018. However, happiness was rated equally positively on the affective component (*M*_affective & positivity_ = 5.47, *se* = 0.04) and the cognitive component (*M*_cognitive & positivity_ = 5.46, *se* = 0.04) Thus, H2 was partially supported.

A significant two-way interaction between country and valence, *F*(3,454) = 15.54, *p* < 0.001, $\eta_{p}^{2}$ = 0.10, not qualified by a 3-way interaction supported H3. Simple effect analyses revealed that Canadians (*M*_Canada & positivity_ = 5.74, *se* = 0.07) and Americans (*M*_US & positivity_ = 5.61, *se* = 0.07) rated happiness to be more positive than Chinese (*M*_China & positivity_ = 5.31, *se* = 0.08) and Koreans (*M*_Korea & positivity_ = 5.20, *se* = 0.06), *Fs* > 12.11, *ps* < 0.022, whereas Koreans (*M*_Korea & negativity_ = 0.86, *se* = 0.06) and Chinese (*M*_China & negativity_ = 0.73, *se* = 0.08) rated happiness to be more negative than Americans (*M*_US & negativity_ = 0.42, *se* = 0.07) and Canadians (*M*_Canada & negativity_ = 0.36, *se* = 0.07), *Fs* > 14.51, *ps* < 0.019. Thus, H3 was fully supported.

### Surprise

The interaction between valence and component, *F*(3,454) = 0.15, *p* = 0.703, was not significant, neither was the three-way interaction, *F*(3,454) = 0.82, *p* = 0.486. We went ahead to test our priori hypothesis. Simple effect analyses revealed that surprise was rated more positively on the cognitive component (*M*_cognitive & positivity_ = 3.67, *se* = 0.05) than on the affective component (*M*_affective & positivity_ = 3.55, *se* = 0.05), *t* = −2.80, *p* = 0.005; however, surprise was also rated more negatively on the cognitive (*M*_cognitive & negativity_ = 2.26, *se* = 0.06) than affective (*M*_affective & negativity_ = 2.18, *se* = 0.06) component, *t* = −1.85, *p* = 0.066. Thus H2 was not supported.

In support of H3, we found a significant two-way interaction between country and valence, *F*(1,454) = 15.93, *p* < 0.001, $\eta_{p}^{2}$ = 0.10, which was not qualified by a three-way interaction, *F*(3,454) = 0.82, *p* = 0.486. Simple effect analyses revealed that Koreans (*M*_Korea & negativity_ = 2.43, *se* = 0.09) and Chinese (*M*_China & negativity_ = 2.38, *se* = 0.11) rated surprise to be more negative compared to Canadians (*M*_Canada & negativity_ = 2.21, *se* = 0.10) and Americans (*M*_US & negativity_ = 1.82, *se* = 0.10), *Fs* > 8.52, *ps* < 0.028 (only Americans were significantly different from Koreans, Chinese, and Canadians), whereas Americans (*M*_US & positivity_ = 4.04, *se* = 0.09), and Canadians (*M*_Canada & positivity_ = 3.87, *se* = 0.09) rated surprise to be more positive than Chinese (*M*_China & positivity_ = 3.34, *se* = 0.10) and Koreans (*M*_Korea & positivity_ = 3.26, *se* = 0.80) *Fs* > 18.57, *ps* < 0.001. Thus, H3 was fully supported.

Finally, we conducted an analysis to eliminate the explanation that the results were due to differences in responses bias between Eastern and Western cultures (see Smith, 2004). We conducted a one-way ANOVA on each of the four pooled response types across all emotions: affective, cognitive, negativity, and positivity, with culture as the predictor (**Table 5**). Results revealed significant differences for three of the pooled variables: affective, *F*(3,454) = 6.86, *p* < 0.001, $\eta_{p}^{2}$ = 0.04 (*post hoc* analyses showed that Americans scored lower than the other three groups); negativity, *F*(3,454) = 6.74, *p* = 0.001, $\eta_{p}^{2}$ = 0.04 (Koreans and Canadians scored higher than Chinese and American); and positivity, *F*(3,454) = 5.46, *p* < 0.001, $\eta_{p}^{2}$ = 0.04 (Koreans scored higher than the other three groups). There were no differences for the pooled cognitive variable, *F*(3,454) = 1.13, *p* = 0.336, $\eta_{p}^{2}$ = 0.01. Although there were differences in three of the pooled variables, the differences were not systematic: the Asian groups scored sometimes higher, sometimes lower, and were sometimes comparable to the North Americans. This suggests that the results were not driven by systematic response biases by any group.

**Table 5:** Means for each variables.

	Affective		Cognitive		Negativity		Positivity	
	*M*	*SD*	*M*	*SD*	*M*	*SD*	*M*	*SD*
Korean	2.91_a_	0.32	3.02_a_	0.33	3.31_a_	0.65	2.62_a_	0.75
Chinese	2.93_a_	0.30	2.97_a_	0.28	3.46_b_	0.58	2.44_b_	0.56
Canadian	2.93_a_	0.23	3.02_a_	0.24	3.62_b_	0.47	2.33_b_	0.43
American	2.77_b_	0.37	2.97_a_	0.31	3.33_a_	0.64	2.41_b_	0.57

*N_Korea_ = 147, N_China_ = 90, N_Canada_ = 109, and N_US_ = 112. Within columns, different subscripts denote significant differences at 0.05.*

## Discussion

To our knowledge, this is the first study that explicitly examined positivity and negativity, separately, of the six basic emotions. Investigating the issue systematically across different cultures, we found that: (1) each emotion indeed contained both positivity and negativity (true for all six emotions); (2) the positivity and negativity ratings were more divergent for affective than for cognitive components (true or partially true for sadness, fear, disgust and anger); (3) American and Canadian ratings were higher for the salient valence and lower for the less salient valence of an emotion, compared to Chinese and Koreans (true or partially true for all six emotions). Thus, the results for most emotions partially or fully supported our hypotheses.

For sadness specifically, the results revealed the delicate aspects of cultural differences in sadness: Easterners (Koreans and Chinese) reported stronger positivity of sadness compared to Westerners (Canadians and Americans), while Westerners reported stronger negativity of sadness compared to Easterners. The finding is consistent with Miyamoto et al. (2014) finding that Asians were more likely than Euro-Americans to believe in motivational and cognitive utility of negative emotions, which mediated cultural differences in emotion regulation. The findings can be attributable to Easterners embracing sadness when they experience it, whereas Westerners feel that they should not have to face sadness (e.g., Spencer-Rodgers et al., 2004).

Regarding fear, only Americans reported stronger cognitive fear, while all others reported stronger affective fear. This suggests that Americans tend to think and conceptualize fear to be negative, while others tend to actually feel it as negative. This provides insight into how people face and deal with fear. In contrast to other cultures, Americans tend to anticipate fear more and feel it less, or perhaps even suppress it (Mauro et al., 1992). Only Koreans reported significantly stronger positivity of fear compared to participants from other countries. Koreans may not view fear as negatively as others due to their cultural background. As a small peninsula country, Koreans have faced (and are still facing) threats to their existence, but despite that, may think they should live their lives as they normally would. China, on the other hand, is the largest country on the continent of Asia, and their ecological and political situation is not threatened by outside factors, which may affect how cultural fear was shaped for Chinese individuals.

Regarding disgust, only Americans reported a stronger level of cognitive disgust relative to others, implying that Americans tend to think and conceptualize disgust as negative, while others tend to feel it more negatively. This suggests that perhaps culture affects how people face and deal with disgust. Americans reported the strongest negativity of disgust, perhaps due to its extreme negativity in American culture. However, for the positivity of disgust, Easterners (Koreans and Chinese) reported stronger positivity of disgust compared to Westerners (Canadians and Americans). This may be due to Easterners’ dialectical thinking style, in that they find both the feeling and thinking of disgust to have benefits for them.

Regarding anger, Easterners (Koreans and Chinese) reported stronger positivity of anger compared to Westerners, especially on the cognitive component. In most countries, anger is viewed as rather negative, but to Koreans (and to some extent, Chinese), anger has a more positive impact. This is consistent with Park et al. (2013) finding that, unlike in the U.S. where Americans with lower social status expressed more anger – likely due to frustration, in Japan, it was those with higher social status that expressed more anger, presumably to display authority. Thus, anger and its expression may have a more positive connotation in East Asian cultures. On the affective component, Americans reported lower negativity than Canadians (and East Asians, to some extent), which was unexpected. It is unclear to what extent this result was due to the fact that the majority of the US sample was Hispanic, whereas the Canadian sample consisted of only Caucasians. Although a within-US comparison revealed no significant differences, the test may have been underpowered. Future research should further investigate this possibility.

Happiness was rated as a positive emotion across all four countries. Still, Westerners reported stronger positivity of happiness compared to Easterners, and Easterners reported stronger negativity of happiness compared to Westerners. Previous findings showed cross-cultural differences in the meaning of happiness and the interpretation of happy images; Westerners typically report happiness to feel uplifting and exciting, but Easterners tend to report happiness as a serene, calm state (Uchida et al., 2004; Tsai, 2007). Not only is happiness considered to evoke less arousal in Easterners compared to Westerners, but happiness also contains possibly more negativity for Easterners (Kubota, 2012). This suggests that happiness may be experienced differently across cultures. To Westerners, perhaps happiness is an emotion that is bright like a clear sunny day, whereas to Easterners, happiness is still positive, but balanced with negativity, like a drizzling sunny day with a rainbow. In other words, how we treat happiness and deal with such an emotion perhaps requires a different attitude for different cultures.

Lastly, although considered a relatively positive emotion, Westerners (Canadians and Americans) reported surprise to be more positive compared to Easterners (Koreans and Chinese). Further, Easterners reported surprise to be more negative compared to Westerners. The results are consistent with the finding that Koreans displayed less surprise than Americans (Choi and Nisbett, 2000). This is perhaps due to Easterners viewing unexpected events to be rather negative despite their dialectical thinking style. This suggests that while Easterners expect changes more so than Westerners (Ji et al., 2001), they may not welcome changes (Ji, 2005). Instead they may be in a pursuit of balance between positivity and negativity, which is consistent with their dialectical thinking style.

Overall, the current research revealed that while there are East versus West cross-cultural differences, each basic emotion has consistent patterns. This is remarkable, considering that although each basic emotion is universally recognized, they are still experienced differently across several cultures. Further, our findings suggest that the degrees of quality of emotions, such as negativity and positivity, and the affective and cognitive constructs of emotion differ across cultures. An implication of this study is that the cognitive processes that arise from culture affect how people feel. This may stem from differences in thinking styles, such as dialectical and linear thinking (Peng and Nisbett, 1999; Ji et al., 2001; Nisbett, 2003). Specifically, Easterners tend to be dialectical when thinking about a situation in a manner that balances the positives and negatives. When things are going well, Easterners might expect a change for the worse, and when things are going badly, they might expect things to get better. This may prepare Easterners for unexpected situations. However, Westerners tend to focus more on one pattern—things will tend to stay as they are, good or bad. This thinking style may lead Westerners to think that things are rather consistent, leading them to concentrate on one side of an issue.

Finally, our results suggest that there is no clear divide separating East from West. For the six basic emotions, the overall patterns were similar across the four countries, such that each of the basic emotions had both positive and negative elements, and that the affective components were stronger (and therefore more polarized) than the cognitive components. The similarities between Koreans and Chinese were stronger than between the US and Canada in some cases (e.g., sadness), but not in others (e.g., anger, where Chinese were more similar to Western cultures than to Koreans). This provides evidence that there are some cultural differences even within Eastern and within Western cultures.

A final implication as a whole include more parsimonious explanations for emotional phenomena in general. For example, there is a large literature on the different kinds of surprise, which is necessary due to the complicated and numerous ways in which surprise has traditionally been conceptualized (see Lorini and Castelfranchi, 2007). A more parsimonious conceptualization of surprise as containing both positivity and negativity (instead of being wholly one or the other) can more easily explain a variety of findings (e.g., surprise as an alarm vs. surprise as a discrepancy between reality and expectation) than requiring multiple conceptualizations.

### Limitation and Future Directions

There are some limitations to the present research. First, participants were all students. While the mean sample age and occupations were consistent across all four countries, we did not sample the general population. As mentioned, the American sample had a higher percentage of Hispanics compared to Caucasians; although the difference between them was not statistically significant, this test may have been underpowered given the amount of participants in the two groups. Thus, it is possible this could have impacted the mixed findings for the Americans.

Next, we only explored the six basic emotions. By using basic emotions, we were able to address nuances in the most common emotions across cultures. That being said, as the basic emotions include more negative than positive emotions, future research should include more positive emotions to test the generalizability of the current findings.

There are known issues of equivalence of meaning across languages and differences in response styles (e.g., Peng et al., 1997; Ji et al., 2000; Smith, 2004). However, acquiescence and extremity are valid representations of cultural differences (Smith, 2004). Thus, even in this case, we argue the results are not simply artefactual, as demonstrated in the supplementary analyses we conducted.

Lastly, we relied on a self-report based methodology. In other future research, it would be interesting to investigate the topic using other approaches, such as behavioral and neuroscience approaches, in order to get a more comprehensive picture of the processes involved in the present findings.

## Conclusion

The goals of the present research were (a) to determine the degree to which each emotion contains both negativity and positivity, and (b) to determine the quality of the differences and similarities of the emotional experience of the basic emotions. The results of the current study supported our hypotheses. The findings revealed that there are rather consistent patterns across Eastern and Western cultures, although differences also exist across cultures, and sometimes even within cultures. This framework is novel, yet compliments existing theory and research. Also, the current paper is the first to examine cultural differences systematically across basic emotions. We hope that this research enhances our understanding of emotion across cultures, as well as provides a new framework with which to examine emotion more generally.

## Ethics Statement

This research was approved by the Queen’s University Institutional Review Board where the study was designed (and also each institution in each country where the studies were conducted), in accordance with APA ethical standards. Written informed consent from each respective country’s participants was given.

## Author Contributions

SA conducted the project across four countries while she was in Canada, analyzed the data, and wrote the paper. L-JJ was a postdoc mentor of SA; shaped the project and contributed to writing the paper. MM conducted the project in the US, and contributed to writing the project. ZZ collected data from China.

## Conflict of Interest Statement

The authors declare that the research was conducted in the absence of any commercial or financial relationships that could be construed as a potential conflict of interest.

## Footnotes

1 Only fear and sadness ratings were similar, *p* = 0.041; for all the other comparisons, *ps* < 0.001.

2 If we use a corrected alpha value, the Korean-Chinese difference will be no longer statistically significant.

3 Again, if we use a corrected alpha, the difference between Koreans-Chinese, and that between Canadians and Americans, will be no longer statistically significant.

4 If we use a corrected alpha value, the difference between Americans and Canadians will be no longer statistically significant.
