# Corrigendum: Two Sides of Emotion: Exploring Positivity and Negativity in Six Basic Emotions across Cultures

**DOI:** 10.3389/fpsyg.2017.01467

**Published:** 2017-09-04

**Authors:** Sieun An, Li-Jun Ji, Michael Marks, Zhiyong Zhang

**Affiliations:** ^1^Department of Psychology, Ashoka University Sonepat, India; ^2^Department of Psychology, Queen's University Kingston, ON, Canada; ^3^School of Psychological and Cognitive Sciences, Peking University Beijing, China; ^4^Department of Psychology, New Mexico State University Las Cruces, NM, United States

**Keywords:** emotion, affect, cognition, culture, dialectical thinking

In the original article, there was a mistake in the legend for Figure 1 as published. There was a transpositional error in the figure. The corrected Figure 1 appears below.

**Figure 1:**
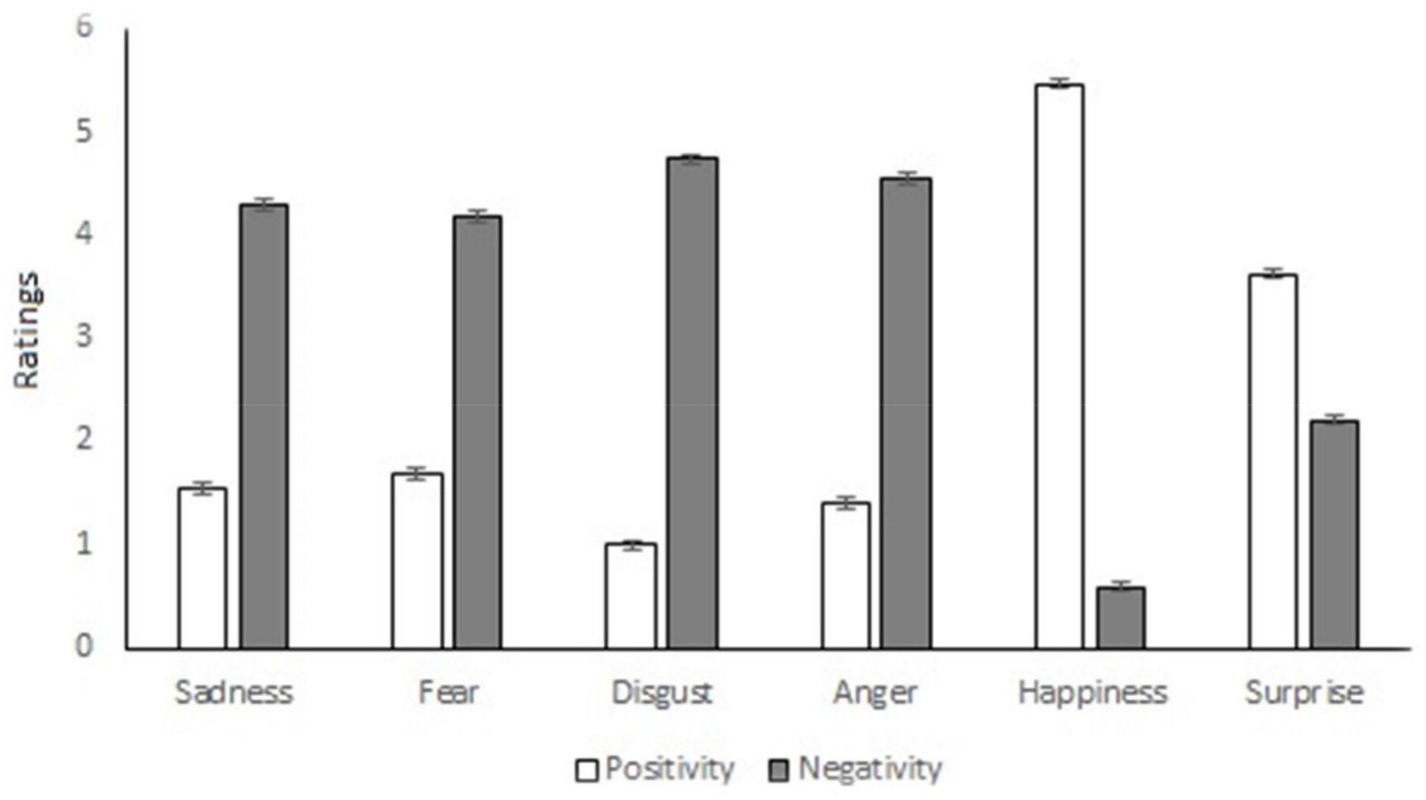
Comparison of the positivity and negativity of emotions (0 = Not at all; 6 = Extremely).

In the original article, there was a mistake in Table 1 as published. The Chinese font for “fear” was smaller than the rest of the font. The corrected Table 1 appears below. The authors apologize for these errors and state that this does not change the scientific conclusions of the article in any way.

**Table 1:** Six Emotions in Korean, Chinese, and English.

**Korean**	**Chinese**	**English**
슬 픔	悲 伤	Sadness
분 노	愤 怒	Anger
혐 오	厌 恶	Disgust
두 려 움	恐 惧	Fear
놀 람	惊 讶	Surprise
행 복	快 乐	Happiness

## Conflict of interest statement

The authors declare that the research was conducted in the absence of any commercial or financial relationships that could be construed as a potential conflict of interest. The reviewer AK and handling Editor declared their shared affiliation.

